# Short and long term representation of an unfamiliar tone distribution

**DOI:** 10.7717/peerj.2399

**Published:** 2016-08-30

**Authors:** Anja X. Cui, Charlette Diercks, Nikolaus F. Troje, Lola L. Cuddy

**Affiliations:** 1 Department of Psychology, Queen’s University, Kingston, Ontario, Canada; 2 Fachbereich Humanwissenschaften, Universität Osnabrück, Osnabrück, Germany

**Keywords:** Statistical learning, Music cognition, Probe tone paradigm

## Abstract

We report on a study conducted to extend our knowledge about the process of gaining a mental representation of music. Several studies, inspired by research on the statistical learning of language, have investigated statistical learning of sequential rules underlying tone sequences. Given that the mental representation of music correlates with distributional properties of music, we tested whether participants are able to abstract distributional information contained in tone sequences to form a mental representation. For this purpose, we created an unfamiliar music genre defined by an underlying tone distribution, to which 40 participants were exposed. Our stimuli allowed us to differentiate between sensitivity to the distributional properties contained in test stimuli and long term representation of the distributional properties of the music genre overall. Using a probe tone paradigm and a two-alternative forced choice discrimination task, we show that listeners are able to abstract distributional properties of music through mere exposure into a long term representation of music. This lends support to the idea that statistical learning is involved in the process of gaining musical knowledge.

## Introduction

Music is ubiquitous in our everyday life. Not only do we actively seek out music, we also encounter it passively, for instance playing softly in grocery stores. Perceiving music involves a diverse range of cognitive processes. These processes give rise to our abilities to recall our favourite songs, appreciate new music, and recognize different music genres. Based on our sense of a specific music genre, we are even able to identify points at which stylistic mistakes are made, e.g., when the music sounds unconventional or “weird.” How do we gain this sense of music? How do we gain a mental representation of a music genre?

For the purposes of this report, “music genre” refers to a category of music the members of which are created using the same conventions, e.g., using the same scales (Fabbri, 1999). For example, our sense of music involves a tacit knowledge of which scale degrees are more important than others in a specific music genre (Krumhansl & Cuddy, 2010). This knowledge has been called our representation of tonal hierarchy, i.e., our mental representation of the rank order of musical tones by stability. Assessing the mental representation of tonal hierarchy may be considered a proxy for the assessment of the representation of a music genre. Tonal hierarchy is a structural principle found in music throughout history and around the world. Some tones are ranked higher than others: These tones occur more frequently, they are rhythmically emphasized, and tend to appear at structurally important positions such as the beginning and ending of a piece.

Assessments of listeners’ representations of tonal hierarchy are highly correlated with an objective feature of music, namely, the statistical distribution of tones (Krumhansl, 1985). Do we gain our sense of music, i.e., a mental representation of the tone distribution, by extracting and abstracting this statistical information from the music to which we are exposed? Recent research has suggested this possibility. Importantly, we may gain our sense of music and become musically enculturated without intending to do so, suggesting that implicit learning plays a substantial role (Tillmann, Bharucha & Bigand, 2000; Bigand & Poulin-Charronnat, 2006; Zatorre & Salimpoor, 2013).

Apart from this top-down explanation of tonal hierarchies, bottom-up mechanisms likely play an important role as well. Recent non-statistical explanations for the tonal hierarchies argue that the frequency of occurrence of certain tones is driven by psychoacoustical similarity between tones, where the similarity could result from virtual pitch class commonality (Parncutt, 2011), or spectral pitch class similarity (Milne, Laney & Sharp, 2015), which in turn then are solidified in a ranking of stability, i.e., the tonal hierarchies. Large et al. (2016) propose a neurodynamic model, in which each tone leaves a trace in an oscillatory neural network. The oscillations interact, creating different patterns of mode-locking. This model proposes that simpler mode-locks, i.e., greater dynamical stability leads to greater tonal stability. This model would explain the high frequency of occurrence of perfect consonances, i.e., dynamically stable tone pairs, in the scales of many musical cultures. The variety of hierarchies found between these cultures over and above the common perfect consonances however makes it unlikely that a pure psychoacoustical explanation can account for the tonal hierarchy (Krumhansl & Cuddy, 2010).

### Implicit and statistical learning

Reber (1967) showed that participants were sensitive to the regularities contained in a stimulus set without having been told about the existence of these underlying regularities. After successfully memorizing a set of four letter sequences generated from a finite-state grammar, participants became increasingly better at memorizing subsequent sets of four letter sequences generated from the same grammar. They also successfully judged whether new stimuli were based on the same regularities. However, participants who were asked to memorize sets of letter sequences formed randomly, i.e., not based on any regularities, did not show an improvement in memorizing new sets. Reber called the acquisition of knowledge about the regularities in a stimulus set without being able to verbalize and without the intention to acquire it, implicit learning. Reber noted the similarity to “perceptual learning” as explored by Gibson & Gibson (1955), but chose to call it “implicit learning” to emphasize the implicit nature of the gained knowledge.

Since then, implicit learning has been investigated in several studies, and also using non-linguistic auditory, musical stimuli (Bigand, Perruchet & Boyer, 1998; Tillmann, 2005; Rohrmeier, Rebuschat & Cross, 2011). In these studies, participants are exposed to stimuli under incidental learning conditions, i.e., without the explicit instruction to acquire knowledge about the underlying regularities of the stimulus set. Afterwards, it is determined whether knowledge has been acquired, and whether this knowledge is implicit or explicit. Successful acquisition of knowledge that participants cannot verbalize and are unaware of is called implicit learning, as was done by Reber (1967); successful acquisition of knowledge that is explicit, i.e., verbalizable by the participant, is called incidental learning (Rohrmeier & Rebuschat, 2012).

Another research area, which also studies knowledge acquisition through exposure was introduced in 1996 by Saffran, Aslin, and Newport. These authors proposed that children learn word boundaries and speech segmentation by mere exposure to statistical regularities contained in speech: Statistical regularities distinguish between more frequently occurring sound sequences forming words and less frequently occurring sound sequences across word boundaries. They demonstrated the successful acquisition of knowledge about the frequency of co-occurrence of syllables found in syllable-triplets presented during exposure, and termed this process “statistical learning.” The basic procedure used in statistical learning research is very similar, if not to say identical, to the procedure used in implicit learning research: Participants are exposed to a stimulus set based on certain regularities and afterwards tested on their reaction to–sometimes new–stimuli based on the same regularities (Saffran et al., 1997; Aslin, Saffran & Newport, 1998).

Differences between the first studies on implicit learning and on statistical learning existed in their focus of interest–grammar learning and speech segmentation respectively–and therefore in their utilized stimuli. Implicit learning research used stimuli based on finite-state grammar, i.e., “sentences” generated based on certain rules, whereas statistical learning research manipulated the statistical information contained in the stimulus set (Saffran, Aslin & Newport, 1996). However, more recently, the similarities between these two lines of research–Reber (1967) had previously attributed a “statistical nature” (p. 856) to his stimuli–have led to the terms implicit learning and statistical learning being used synonymously (Perruchet & Pacton, 2006; Rohrmeier & Rebuschat, 2012). For the purposes of this report, we will use the term statistical learning, as we manipulated the statistical information of our stimuli.

### Statistical learning of music

Statistical learning may be involved in helping us gain our sense of music, as it may take place without the intention to acquire knowledge, and different types of studies have suggested that we form our sense of music through passive exposure. Cross-cultural research using stimuli as diverse as Western tonal music, traditional North Indian music, or traditional Balinese music shows that participants are more attuned to the statistical distribution of tones in a given music genre if they are familiar with it than if they have had no prior exposure (Castellano, Bharucha & Krumhansl, 1984; Kessler, Hansen & Shepard, 1984). Developmental studies show a more detailed representation of tonal hierarchy, and thus the statistical distribution of tones, with increasing age and music training (Trainor & Trehub, 1992; Trainor et al., 2012).

Recent research has extended statistical learning research, first conducted using linguistic stimuli (Saffran, Aslin & Newport, 1996; Saffran et al., 1997; Aslin, Saffran & Newport, 1998), to include non-linguistic auditory, musical stimuli, i.e., tones. This research asks: Can participants extract and abstract statistical regularities contained in musical stimuli to which they are exposed? Participants have been shown to be able to learn about co-occurrences of tones in tone-triplets (Saffran et al., 1999), and timbres in timbre-triplets (Tillmann & McAdams, 2004), as well as non-local dependencies in sequences of eight tones (Kuhn & Dienes, 2005), and finite-state grammar based tone sequences (Loui, Wessel & Hudson Kam, 2010). The study by Kuhn & Dienes (2005), by employing stimuli based on non-local dependencies, especially provides evidence that participants are abstracting statistical regularities rather than merely abstracting local chunks of information, i.e., chunks of three tones when using triplets. As these authors used non-local dependencies, participants were not able to chunk material together.

Among the different ways to assess statistical learning, we consider the following two the most revealing. After exposure to the stimulus set, i.e., music or a number of music-like sequences, which puts forward the statistical regularities which the participant is supposed to learn, the participant may be asked in a two-alternative forced-choice task which of two stimuli seems more familiar: one that is created using the same statistical regularities of the exposed stimulus set, or one that is created using different statistical regularities. The more prevalent choice of the stimulus created using the same statistical regularities is considered an indicator of statistical learning.

Alternatively, a measure of tonal “fit” may be obtained using the probe tone method, introduced by Krumhansl & Shepard (1979). The probe tone method asks participants to rate how well a probe tone fits into a previously presented probe tone context. The obtained ratings serve as a measure of a participant’s representation of the tonal hierarchy of the musical system defined by the probe tone context. A variety of stimuli can be used as probe tone contexts: scales, chords, or fragments of tone sequences (Krumhansl, 1990; Krumhansl & Kessler, 1982). The probe tone method has been used in studies of statistical learning to assess participants’ sensitivity to the statistical regularities of novel musical stimuli to which they have been exposed (Loui, Wessel & Hudson Kam, 2010). Assuming successful acquisition of the statistical distribution of tones found in the exposed stimuli, ratings of probe tones following a tone sequence created with the same statistical regularities as the exposed stimulus set should reveal that tones occurring more often in the exposed stimulus set are rated as more fitting than less frequently occurring tones.

### Our experiment

Here, we report on an experiment conducted to expand on this body of research. Several studies have investigated statistical learning of music by assessing abstraction of sequential rules (Saffran et al., 1999; Tillmann & McAdams, 2004; Kuhn & Dienes, 2005; Loui, Wessel & Hudson Kam, 2010). However, since it is thought that our sense of a music system may be based, in part, on the abstraction of the *distribution* of tones (Krumhansl & Cuddy, 2010), we wanted to investigate statistical learning of music by assessing abstraction of a distribution. A study by Cui et al. (2015) suggests that people are able to abstract distributional information. In this study, participants were exposed to tone sequences created using a tone distribution, and afterwards asked to select the more familiar tone sequence in a two-alternative forced-choice task. Participants chose the tone sequence based on the same tone distribution more often than the tone sequence based on another tone distribution. Here, we aim to expand this research by assessing statistical learning with both a two-alternative forced-choice task and by comparing probe tone ratings obtained before and after an exposure phase.

In the probe tone paradigm, participants are usually asked to indicate goodness of fit of probe tones on a 7-point Likert scale (Krumhansl & Cuddy, 2010). However, response styles may differ between participants, such that some participants tend to choose more extreme answers while others gravitate towards the center of the response scale, shown in general for instance by Hui & Triandis (1989), or Chen, Lee & Stevenson (1995), and also in studies using the probe tone paradigm (Krumhansl & Shepard, 1979; Cuddy & Badertscher, 1987). In the study by Cuddy & Badertscher (1987), participants with a high level of music training used a wider range of the 7-point Likert scale than participants with less music training. In an effort to control for these differences, we did not ask *how well* but *whether* a probe tone fits, i.e., instead of asking for a response on a 7-point scale, we asked for “yes” or “no” responses. In order to obtain a graded response profile for the different probe tones we included multiple trials for each probe tone, and regarded the number of times each probe tone was classified “fitting” as a probe tone rating.

Our stimuli were based on a whole-tone scale. This choice was made in an effort to ensure that the artificial “music genre” was unfamiliar to participants. We assumed that participants had primarily been exposed to Western music, which is based on diatonic scales. Listeners have been shown to be sensitive to the distributional information contained in music even if this music is unfamiliar (Oram & Cuddy, 1995; Vuvan, Prince & Schmuckler, 2011; Lantz, Kim & Cuddy, 2014; Raman & Dowling, 2016). This pattern of result could arise if listeners have a short term memory for the specific stimulus used to elicit the probe tone ratings without possessing a long term memory for the music genre itself (Parncutt, 2011). Therefore, we came up with a novel experimental design allowing us to differentiate between this general sensitivity to distributional information contained in a probe context–which we call short term representation of the tone distribution here–from an actual abstraction of the characteristic distribution of a music genre–which we call long term representation of the tone distribution. We assume that even though a probe tone context may invoke a music genre, its tone distribution does not necessarily have to correspond to the tone distribution of the music genre.

For this purpose, we exposed participants to tone sequences created using carefully manipulated distributions. Before and after an exposure phase, in which participants listened to an exposure stimulus, we obtained probe tone ratings. By using a distribution for probe tone contexts to elicit these ratings that was highly similar but not identical to the distribution for the exposure stimulus, we were able to formulate exact hypotheses regarding the probe tone ratings. The similarity between the two distributions was intended to suggest to the participants that tone sequences heard as probe tone contexts and the exposure stimulus belonged to the same music genre. We expected (a) that participants would exhibit sensitivity to the distributional information contained in the probe tone contexts themselves, but also (b) that after exposure, participants would differentiate tones that were part of the music genre but did not occur in probe tone contexts from tones that were neither part of the music genre nor tones in probe tone contexts. Thus, our stimuli allowed us to formulate exact hypotheses regarding the probe tone ratings depending on whether participants have a short term representation of the tone distribution, and furthermore, whether participants have successfully gained a long term representation of the tone distribution after the exposure phase.

## Methods

### Participants

Participants were 29 female and 11 male students from various departments who received monetary compensation. The average age was *M* = 21.13 years, *SD* = 3.05 years. The average number of years of music training was *M* = 8.18 years, *SD* = 3.98. None of the participants claimed to possess perfect pitch. Further descriptive statistics of our sample can be found in the Supplemental Information 1.

### Procedure

The procedure was approved by the General Research Ethics Board of our institution (“GPSYC-720-15 Acquisition of Musical Knowledge”; ROMEO # 6016140). The experiment comprised four parts: pre-exposure probe tone ratings, exposure, post-exposure probe tone ratings, and a two-alternative forced-choice discrimination task. Tone sequences heard during these parts were generated based on statistical regularities, which are explained later on in this section (see ‘Stimuli’). After obtaining written consent from the participant, he or she was asked to sit in front of a 21.5″ monitor (Dell E2214H) and wear earphones ($ETYM\bar {O}TIC$ mkIsolator^™^). Participants were instructed to wear the earphones throughout the experiment and to follow the instructions on the screen. The stimuli were presented using E-Prime 2.0, running on a personal computer (Dell Optiplex 7020).

Before and after exposure, participants were presented with probe tone contexts immediately followed by a probe tone. They were then asked, whether the last tone of the tone sequence, i.e., the probe tone, fit the rest of the melody. There were 160 trials both before and after exposure. These phases each took about 20 min. During exposure, they were exposed to a continuous stream of tones generated from an underlying tone distribution (referred to as exposure sequence in the remainder of this report). During this time, participants were instructed to fill out questionnaires containing questions about demographic information and music training history and music interaction styles. The average responses on the Music Engagement Questionnaire (Vanstone et al., 2015), Short Test of Music Preferences (Rentfrow & Gosling, 2003), and Goldsmiths Musical Sophistication Index (Müllensiefen et al., 2014) can be found in the appendix. This phase took 30 min. At the end of the experiment, participants were presented with 40 pairs of tone sequences. Participants were asked to indicate, which of the two tone sequences they found more familiar. This phase took about 10 min.

### Stimuli

All tone sequences heard during the experiment were constructed based on a distribution forming the basis of an unfamiliar music genre. To minimize similarity to standard Western music, the music genre was based primarily on a whole-tone scale, i.e., a non-diatonic scale. The whole-tone scale uses tones separated by a whole-tone, and thus can either be constructed using the tones C, D, E, F♯, G♯, and A♯, or C♯, D♯, F, G, A, and B.

We created four tone categories. Membership of a specific tone in a tone category is defined by two criteria: whether it occurs in the exposure sequence (in the exposure sequence: *E*; not occurring in the exposure sequence: $\bar {E}$), and whether it occurs in the probe tone contexts (occurring in probe tone context: *P*; not occurring in probe tone context: $\bar {P}$). Tones either occurred in the exposure sequence and in probe tone contexts (*EP*), in the exposure sequence but not in the probe tone context ($E\bar {P}$), not in the exposure sequence but in the probe tone context ($\bar {E}$), or in neither the exposure sequence set nor probe tone context ($\overline{EP}$).

**Figure 1:**
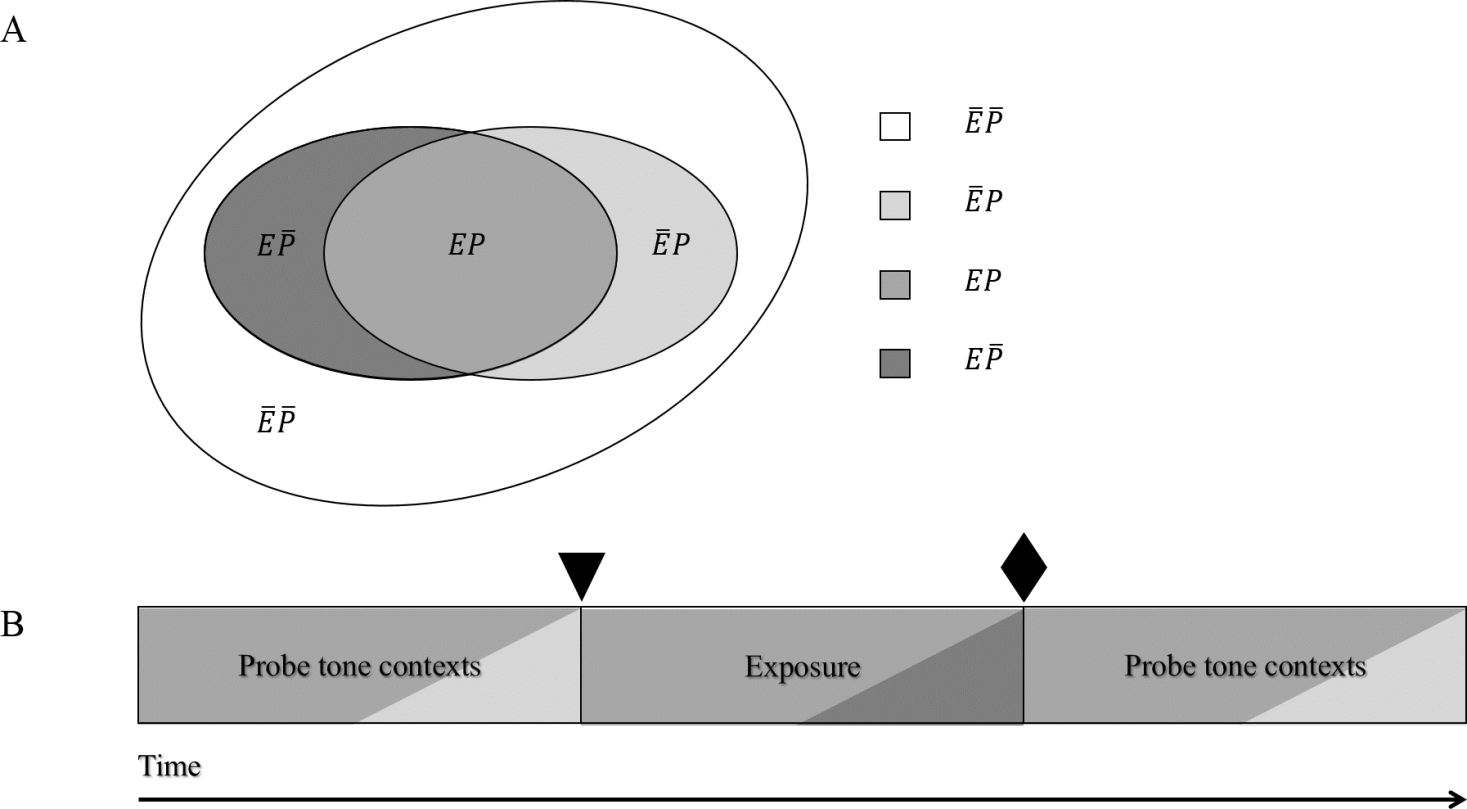
(A) Underlying structure of stimuli: tones belonged to one of four tone categories. (B) Tone categories heard throughout the experiment: participants have heard tones belonging to categories *EP* and $\bar {E}P$, and also $E\bar {P}$. The shadings in Fig. 1B correspond to the respective shadings in Fig. 1A. Thus, for example, probe tone contexts contained mainly tones of probe tone category *EP* but also some tones of probe tone category of probe tone category $\bar {E}P$. The triangle marks the point at which participants have heard tones belonging to categories *EP* and $\bar {E}P$; the diamond marks the point at which participants have heard tones belonging to categories *EP* and $\bar {E}P$, and also $E\bar {P}$.

Figure 1A visualizes the structure underlying our stimuli. The four different colours correspond to one probe tone category each. The genre consists of tones falling into the grey, the dark grey, and the light grey areas, with the bulk of the genre consisting of tones falling into the grey area. Tones falling into the white area were only heard as probe tones but not as part of any probe tone contexts or in the exposure sequence. Figure 1B illustrates which tone categories were heard throughout the experiment. After the first probe tone test, but before exposure (marked in Fig. 1B by a triangle), participants have heard tones belonging to categories *EP* (grey) and $\bar {E}P$(light grey), after exposure (marked in Fig. 1B by a diamond), participants have heard tones belonging to categories *EP* (grey) and $\bar {E}P$ (light grey), and also $E\bar {P}$(dark grey) Thus, it is only after exposure that participants have heard tone sequences containing all tones that belong to the unfamiliar music genre.

Tones in tone category *EP* formed the basis of and occurred most frequently in the unfamiliar music genre. These were the tones of a whole-tone scale. Two other tones each were selected to form tone categories $E\bar {P}$, $\bar {E}P$ and $\overline{EP}$. Probe tone contexts contained the tones C4, D4, D♯4, E4, F♯4, G♯4, A4, and A♯4, which occurred with probabilities of .147, .147, .06, .147, .147, .147., .06, and .147, respectively. The exposure stimulus contained the tones C4, C♯4, D4, E4, F♯4, G4, G♯4, and A♯4, which occurred with probabilities of .147, .06, .147, .147, .147, .06, .147, and .147, respectively. As 88% of the tones heard in probe tone contexts and in the exposure sequence were tones in tone category *EP*, these tone sequences can be regarded as highly similar, and thus as belonging to the same music genre. Probe tone responses were elicited to tones from all four tone categories in 40 trials per tone category both before and after the exposure phase, yielding 320 probe tone responses in total. Table 1 details which tones were in each tone category.

**Table 1:** Tones in each tone category.

Tone category	Tone
*EP*	C4, D4, E4, F♯4, G♯4, A♯4
$\bar {E}P$	D♯4, A4
$E\bar {P}$	C♯4, G4
$\overline{EP}$	F4, B4

All tone sequences were generated in MATLAB using recordings of single tones on a Steinway & Sons grand piano B model from the online archive of the University of Iowa Electronic Music Studios (Fritts, 2013). Tones in tone sequences were all 150 ms long. These tones were randomly ordered into sequences used as probe tone contexts, such that each probe tone context contained 30 tones belonging to tone category *EP* and four tones belonging to tone category $\bar {E}P$ (see above for probabilities of each tone). To prevent expectations about the length of each stimulus which could imply an underlying rhythmic structure, one tone was cut from 25% of the tone sequences, and one tone was added to another 25% of the tone sequences. This tone was randomly selected from the eight tones that could occur in probe tone contexts. Each probe tone context was immediately followed by a probe tone, which lasted 800 ms. To form the exposure sequence, 12,000 tones were randomly ordered such that the majority of the tones were tones belonging to tone category *EP* and some of the tones belonged to tone category $\bar {E}P$ (see above for probabilities of each tone). The exposure sequence took 30 min. Most participants took the entire 30 min to fill out the questionnaires, and were instructed to either answer the remaining questions before continuing with the rest of the experiment or continued with the rest of the experiment without answering the remaining questions. For those, who had filled out the questionnaire in its entirety before the sequence had ended, the experimenter invited the participants to doodle on their phones while the rest of the sequence played (this invitation was accepted by all of these participants).

For the discrimination task, 40 pairs of tone sequences were generated. Each tone sequence contained 34 randomly ordered tones. For one tone sequence of each pair, 30 tones were selected from the tones C4, D4, E4, F♯4, G♯4, and A♯4, and four tones were selected from the tones C♯4, and G4, thus resembling in its distribution the continuous tone stream heard during the exposure phase (see Table 1). For the other tone sequence in each pair of tone sequences, 30 tones were selected from the tones C♯4, D♯4, F4, G4, A4, and B4, and four tones were selected from the tones F♯4, and G♯4, thus being dissimilar to any of the tone sequences heard before the discrimination task.

### Analysis

The number of times a participant had indicated that the probe tone fit for each probe tone category was regarded as a probe tone rating for that category by that participant. Since participants responded either “yes” or “no,” the task was essentially a binary choice-task, preventing the analysis of the data using a standard ANOVA as the assumption of the normal distribution of mean values is violated. Instead, we analyzed the data using a mixed-effects model in MATLAB (fitglme). This analysis allows specification of the underlying, binomial response distribution.

In our model, we included the random effect of “Participant.” “Exposure” (*E*, $\bar {E}$), “Probe Tone Context” (*P*, $\bar {P}$), “Time” (before exposure, after exposure), and interactions between these predictors were included as fixed effects in the model. We expected the three-way interaction Time, Exposure, and Probe Tone Context to be a significant predictor, such that probe tone category $E\bar {P}$, i.e., tones occurring in the exposure stimulus (Exposure = *E*) but not in probe tone contexts (Probe Tone Context = $\bar {P}$), receive higher ratings after the exposure phase (Time = after exposure).

To investigate specific hypotheses we carried out additional analyses:

(1) Probe tone ratings obtained before exposure were analyzed with a mixed-effects model including the random effect of “Participant.” “Exposure” (*E*, $\bar {E}$), “Probe Tone Context” (*P*, $\bar {P}$), and interactions between these predictors were included as fixed effects in the model. Here we expect a significant effect of Probe Tone Context, such that tones occurring in the probe tone context (*P*) would receive higher probe tone ratings, as only tones of probe tone categories $\bar {E}P$ and *EP* occur in probe tone contexts, and thus are heard before exposure.
(2) As tones in probe tone category $\bar {E}P$ occur less often than tones in tone category *EP*, we expected to see a significant difference between probe tone ratings for these two categories. Probe tone ratings for probe tone categories $\overline{EP}$ and $E\bar {P}$ however should not differ. These contrast analyses were carried out using hypothesis tests on the appropriate fixed effects of the mixed-effects model. This analysis complements the preceding analysis to investigate whether participants have a short term representation of the tone distribution.
(3) A third mixed-effects model was designed to analyze probe tone ratings obtained for probe tones that did not occur in probe contexts, investigating whether participants would develop a long term representation of the tone distribution after exposure. Besides the random effect of “Participant,” “Exposure” (*E*, $\bar {E}$), “Time” (before exposure, after exposure), and interactions between these factors were included as fixed effects in the model. Tones of probe tone category $E\bar {P}$ were heard during exposure but tones of probe tone category $\overline{EP}$ were not. Thus, we expected a significant interaction of Exposure and Time such that probe tone ratings for tones that occur during exposure, $E\bar {P}$, would increase after exposure, but probe tone ratings for tones that do not occur during exposure, $\overline{EP}$, would not increase after exposure. Additional mixed-effects models analyzing probe tone ratings for each of these categories with the random effect of “Participant” and the fixed effect of “Time” (before exposure, after exposure) were also planned to examine this interaction. One hypothesis test on the fixed effect of “Exposure” was conducted to determine whether probe tone ratings for probe tone category $E\bar {P}$ and probe tone ratings for probe tone category $\overline{EP}$ differed before exposure. A similar analysis examined whether the probe tone ratings for these two categories differed after exposure.

Average response times for each probe tone category, for which we can assume a normal distribution, were analyzed with a standard ANOVA with three within subject variables (Time: before exposure, after exposure; Exposure: *E*, $\bar {E}$; Probe Tone Context: *P*, $\bar {P}$) to help determine if there were training effects. If participants merely understood the task better after the exposure phase (Bigand & Poulin-Charronnat, 2006), this should be reflected in lower reaction times, i.e., a main effect of Time.

From participants’ responses in the two-alternative forced-choice discrimination task we calculated the proportion of trials in which participants selected the tone sequence resembling the exposure phase tone sequence over the other tone sequence, which was dissimilar to any of the tone sequences heard prior to the discrimination task. The choice of the tone sequence resembling the tone stream heard during the exposure phase was classified as “correct”. Successful learning should be reflected in a “percent correct” significantly different from percent correct = .50. See Data S1 for a file containing the data.

## Results

**Figure 2:**
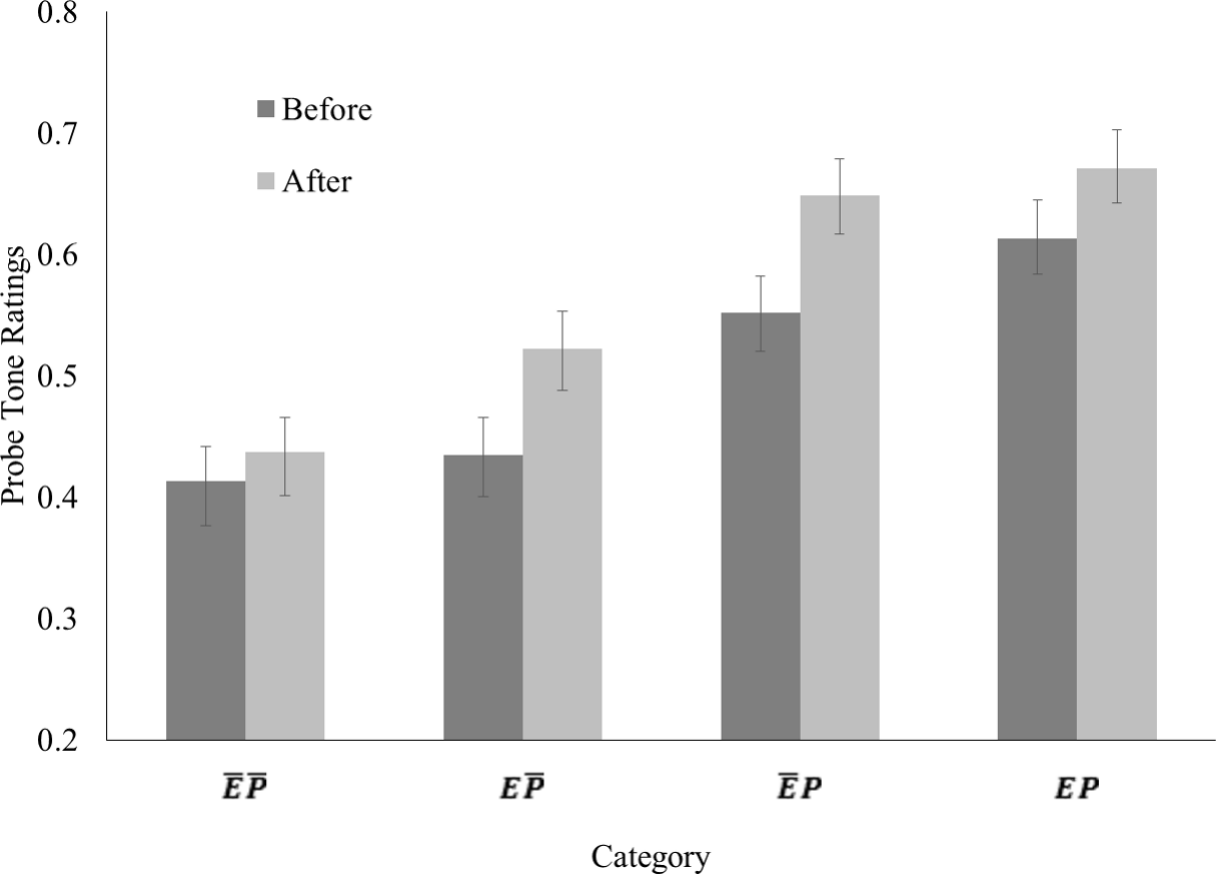
Average probe tone ratings, i.e., proportion of “yes” responses in trials, for each probe tone category. Error bars depict the standard error of mean.

The mixed-effects model of all probe tone ratings was able to explain 59% of the variability in the responses, *R*^2^ = .59. Several predictors were significant: Time, *t*(312) = 7.94, *p* < .001, Exposure, *t*(312) = 5.61, *p* < .001, and Probe Tone Context, *t*(312) = 19.76, *p* < .001. Additionally, the three-way interaction of Time, Exposure, and Probe Tone Context was a significant predictor, *t*(312) = − 2.89, *p* = .004. None of the other included fixed effects were significant, *p*s > .05. The probe tone ratings for each category, before and after exposure, are depicted in Fig. 2. As can be seen here, ratings were higher for tones occurring in the probe tone contexts (*P*), tones occurring during exposure (*E*), and after exposure. More specifically, tones occurring in the exposure stimulus (Exposure = *E*) but not in probe tone contexts (Probe Tone Context = $\bar {P}$), receive higher ratings after the exposure phase (Time = after exposure) as predicted by our hypotheses.

We followed up the significant three-way interaction with additional analyses, as outlined above. Probe tone ratings obtained before exposure, depicted in Fig. 2 as dark grey bars, were entered into a second mixed-effects model. The predictor Probe Tone Context was significant, *t*(156) = 13.00, *p* < .001, such that tones occurring in the probe tone context yielded higher probe tone ratings as expected. However, the predictor Exposure was also significant, *t*(156) = 3.47, *p* = .001. The interaction of Probe Tone Context and Exposure was not a significant predictor, *p* > .05. Contrast analyses between the different probe tone ratings revealed that the effect of Exposure was driven by a significant difference between ratings for probe tone category *EP* and ratings for probe tone category $\bar {E}P$, *F*(1, 156) = 13.08, *p* < .001. The difference between ratings for probe tone categories $E\bar {P}$ and $E\bar {P}$ was not significant, *p* > .05.

The third mixed-effects model analyzing probe tone ratings obtained for probe tones, which did not occur in probe tone contexts, revealed that all fixed effects were significant predictors: Time, *t*(156) = 4.74, *p* < .001, Exposure, *t*(156) = 4.53, *p* < .001, as well as their interaction, *t*(156) = 2.65, *p* = .009. As already mentioned, the difference between ratings for probe tone categories $\bar {E}$ $\bar {P}$ and $E\bar {P}$ was not significant before exposure, *p* > .05. However, the difference between these ratings was significant after exposure, *F*(1, 156) = 25.75, *p* < .001, such that ratings for probe tone category $E\bar {P}$ were higher than ratings for probe tone category $\overline{EP}$. Mixed-effects models for probe tone ratings of each of these two categories showed that the predictor Time was significant probe tone category $E\bar {P}$, *t*(78) = 5.25, *p* < .001, such that ratings for probe tone category $E\bar {P}$ were higher after exposure, but not significant for probe tone category $\overline{EP}$, *p* > .05,

The ANOVA on the reaction time revealed a significant main effect of Probe Tone Context, *F*(1, 39) = 8.83, *p* = .005, but no significant main effect of time, no significant main effect of Exposure, and no significant interactions, *p*s > .05. Reaction times were longer for probe tones, which did not occur in probe tone sequences, *M* = 1183.30 ms, *SD* = 556.19 ms, than for probe tones, which occurred in probe tone sequences, *M* = 1054.18 ms, *SD* = 465.51 ms.

Analysis of participants’ performance in the discrimination task revealed a percentage of correct answers, which was significantly higher than chance (.50), *M* = .75, *SD* = .20, as determined by a one sample *t*-test, *t*(39) = 7.71, *p* < .001. There were four participants whose performance at the discrimination task was at 100%. The correlation between participants’ performance and years of music training was not significant, *r*(37) = − .17, *p* > .05.

## Discussion

Corresponding to our hypotheses, we found that the three-way interaction of Time, Exposure, and Probe Tone Context was a significant predictor for probe tone ratings in our mixed-effects model. Additional analyses were carried out to address specific hypotheses, as specified above (see ‘Analysis’). A second mixed-effects model on probe tone ratings obtained before exposure revealed that the predictor Probe Tone Context was significant, such that probe tones occurring in probe tone contexts ($\bar {E}P$ and *EP*) received higher probe tone ratings than probe tones, which did not occur in probe tone contexts ($\overline{EP}$ and $E\bar {P}$). The predictor Exposure was also significant, although subsequent contrast analyses showed that this was driven by a significant difference between probe tone ratings for probe tone category $\overline{EP}$ and *EP*, while the difference between probe tone ratings for probe tone category $\overline{EP}$ and $E\bar {P}$ did not differ significantly. Thus, this differentiation of probe tones suggests that participants were sensitive to the distributional information contained in the short tone sequences used as probe tone contexts. The rank in probe tone ratings corresponds to the relative frequency with which these tones occurred in probe tone ratings (lowest ratings for $\overline{EP}$ and $E\bar {P}$, which never occurred, higher rating for $\bar {E}P$, which occur, and highest rating for *EP*, which occur most often), corroborating this conclusion.

This finding is in line with results from several studies demonstrating the sensitivity to distributional information in tone sequences, even if the music genre underlying these tone sequences is unfamiliar (Oram & Cuddy, 1995; Lantz, Kim & Cuddy, 2014; Raman & Dowling, 2016). This result also strongly suggests that the modified version of the probe tone paradigm used in our experiment, which asks *whether* the probe tone fits, is an equivalent to the paradigm introduced by Krumhansl & Shepard (1979), which asks *how well* the probe tone fits. We modified the probe tone paradigm in an effort to control for between subject variance in response styles, either due to general response styles or due to an influence of music training on the range of responses that are given by a participant (Krumhansl & Shepard, 1979; Cuddy & Badertscher, 1987; Hui & Triandis, 1989; Chen, Lee & Stevenson, 1995). We suggest that this modified version of the probe tone paradigm presents a task that is easier to follow than the original probe tone paradigm, ameliorating concerns raised by Bigand & Poulin-Charronnat (2006) about the influence of task difficulty on task performance. Results from an experiment using both the Likert scale version and our version of the probe tone paradigm to elicit ratings from the same stimuli should be compared directly to assess this methodological modification in more detail.

A third mixed-effects model analyzing probe tone ratings obtained for probe tones that did not occur in probe tone contexts, revealed that the interaction of Time and Exposure was a significant predictor, such that probe tone ratings were similar for categories $E\bar {P}$ and $\overline{EP}$ before exposure, but differed after exposure. Specifically, we found increased probe tone ratings for probe tone category $E\bar {P}$ after exposure, but not for probe tone ratings for probe tone category $\overline{EP}$. The absence of a main effect of time in the factorial ANOVA carried out on the reaction times for the probe tone responses shows that any difference between probe tone ratings before and after the exposure phase is unlikely due to a better grasp of the experimental task. Had participants come to understand the task better over the course of the experiment, we would have expected decreased reaction times for the probe tone responses obtained after the exposure phase. Importantly, tones in probe tone category $E\bar {P}$, while belonging to the music genre, only occurred in the exposure tone sequence and did not occur in the probe tone contexts. Thus, probe tone contexts presented after the exposure phase defined a musical system, whose representation included tones in probe tone category $E\bar {P}$. Therefore, participants must have successfully abstracted the distributional information presented during the exposure phase and integrated it with distributional information contained in probe tone contexts to form a long term representation of the tone distribution of the musical genre.

Participants were not instructed to learn about the unfamiliar music system, and furthermore, were asked to fill out questionnaires during the exposure phase, rendering the music listening during this phase passive. This suggests that listeners may gain a mental representation of the music genre through mere exposure. As we cannot comment on whether participants could verbalize the knowledge that they gained we conclude that participants showed at least successful incidental learning and potentially implicit learning (Rohrmeier & Rebuschat, 2012).

This conclusion is also supported by the performance of participants in the two-alternative forced-choice discrimination task completed at the end of the experiment. Participants indicated that tone sequences, which resembled the exposed music genre, seemed more familiar than tone sequences, which did not resemble the exposed music genre. Four participants even exhibited perfect discrimination between the two types of tone sequences. This performance is remarkable when considering that tone sequences presented during the discrimination task were all based on whole-tone scales. One tone sequence was based on the same tone distribution underlying the exposure sequence, using the C4, C♯4, D4, E4, F♯4, G4, G♯4, and A♯4, with probabilities of occurrence of .147, .06., .147, .147, .147, .06, .147, and .147, respectively. The other tone sequence used the tones C♯4, D♯4, F4, F♯4, G4, G♯4, A4, and B4, with probabilities of occurrence of .147, .147, .147, .06, .147, .06, .147, .147, respectively. Thus, the intervals occurring in each tone sequence would be similar, with prevalent intervals such as major second, major third, tritone, minor sixth, and minor seventh. Discrimination between the two tone sequences would not be helped greatly by abstraction of sequential rules, such as one based on intervals or chunks of two tones. Therefore, our findings lend further support to the idea that passive statistical learning of distributional information is involved in the gaining of musical knowledge (Castellano, Bharucha & Krumhansl, 1984; Kessler, Hansen & Shepard, 1984; Trainor & Trehub, 1992; Tillmann, Bharucha & Bigland, 2000; Bigand & Poulin-Charronnat, 2006; Trainor et al., 2012; Zatorre & Salimpoor, 2013).

Interestingly, there was no relationship between participants’ performance in the discrimination task and the years of music training that they had received. This suggests that statistical learning might be a cognitive capacity that is readily not influenced by music training. Statistical learning was initially studied using auditory linguistic stimuli (Saffran, Aslin & Newport, 1996; Saffran et al., 1997; Aslin, Saffran & Newport, 1998), but has also been shown in the visual domain (Kirkham, Slemmer & Johnson, 2002). We might therefore conjecture that statistical learning is a general cognitive capacity employed to make sense of a variety of stimuli, and is not readily influenced by training in one specific area, in which statistical learning might be employed.

Most studies conducted to study the statistical learning of musical material have used stimuli based on sequential rules (Bigand, Perruchet & Boyer, 1998; Saffran et al., 1999; Tillmann & McAdams, 2004; Kuhn & Dienes, 2005; Loui, Wessel & Hudson Kam, 2010). Given that the mental representation of music correlates with *distributional* properties of music (Krumhansl & Cuddy, 2010), we wanted to investigate whether participants are able to abstract distributional information contained in tone sequences to form a mental representation. Our findings show that indeed, listeners are able to abstract this information into a long term representation.

The results presented here corroborate the conclusion drawn by Cui et al. (2015): The passive statistical learning of the frequency of occurrence of tones, i.e., a distributional rule, a structural principle of music (Krumhansl & Cuddy, 2010), could be a model for the process of gaining a mental representation of a music genre. We suggest that people gain a mental representation of music through mere exposure by extracting and abstracting the distributional information contained in the music. The mental representation of this distributional information is updated, when music whose underlying distribution resembles this mental representation is encountered, such that novel information can be incorporated into the mental representation.

In future studies, then, different manipulations could aim at either preventing update of the mental representation, or mimicking regular encounters with music, i.e., enabling regular updates of the mental representation. A potential manipulation could involve the change of the tone sequences heard during the exposure phase such that they do not resemble the tone sequences heard as probe tone contexts. We would assume that participants do no update the mental representation, and thus that probe tone ratings elicited before and after the exposure phase do not show differences.

Another avenue of interest is the longevity of the gained mental representations. For how long after the experiment has ended will participants retain the mental representation? We encounter music throughout our everyday lives, which ensures that our mental representation of the dominant music system around us can be updated and maintained on a regular basis. This then results in mental representations of tonal hierarchy that can be elicited even with probe tone contexts with sparse distributional information (Krumhansl & Kessler, 1982; Vuvan, Prince & Schmuckler, 2011). An experiment manipulating the time elapsed between the exposure to an unfamiliar music genre and the assessment of the mental representation of the tone distribution of this genre would elucidate this question.

Our account of the process underlying the gaining of a mental representation of music would explain why listeners exhibit a more detailed representation of the tonal hierarchy with increasing training and age (Cuddy & Badertscher, 1987; Trainor & Trehub, 1992; Trainor et al., 2012). These listeners have presumably had more exposure to music, i.e., more opportunity to update the mental representation of the tone distribution underlying the music, leading to a clearer distinction between tones occurring with different frequencies. Trainor & Trehub (1992) found for instance, that adults included more of the diatonic tones in their representation of the Western diatonic music system than children. Trainor et al. (2012) proposed that active music making is a driving force in obtaining gaining our representation of tonal hierarchy. Based on our experiment we might wonder how much of the effect ascribed to active music making can be explained by the additional exposure to the music that music-making participants receive compared to non music-making peers. In a similar vein, listeners, who have never been exposed to a foreign music genre, are still sensitive to the distributional information in probe tone contexts (Castellano, Bharucha & Krumhansl, 1984; Kessler, Hansen & Shepard, 1984). However, those listeners, who have been exposed to this music genre, have been able to update and maintain their mental representation, thus displaying heightened sensitivity to the distributional information of the music genre itself.

In conclusion, we have shown here that listeners are able to abstract the distributional information contained in tone sequences, and gain a long term representation of the tone distribution after short exposure to an unfamiliar music system. Our results support the idea that statistical learning is involved in helping us abstract the distribution of tones characteristic for a music system, and thereby gain a sense for that music system. Future studies investigating how this mental representation can be used to distinguish between music systems and the longevity of this mental representation would further expand our understanding of this process.

##  Supplemental Information

10.7717/peerj.2399/supp-1 Supplemental Information 1 Further descriptives of sample

10.7717/peerj.2399/supp-2 Data S1 Behavioural data and participant descriptives

## Additional Information and Declarations

### Data Availability

The following information was supplied regarding data availability: The raw data has been supplied as Supplemental Information.
