# Supplementary material for: Short and long term representation of an unfamiliar tone distribution
Source: PeerJ. 2016 Aug 30;4:e2399. doi: 10.7717/peerj.2399 (PMC5012311; doi:10.7717/peerj.2399)
Supplement: Supplemental Information 1 [file peerj-04-2399-s001.docx]

# Supplemental File: Further Descriptives

The Music Engagement Questionnaire (Vanstone et al., 2015) measures engagement with music in daily life. It comprises six subscales: music in daily life, emotional listening experience, musical performativity, musical consumer behaviour, responsive music listening, musical preference. These factors are assessed using 35 statements. Participants are asked to rate how well each statement describes themselves on a scale from 1 (“Not at all”) to 5 (“Very much”). The values in Table 1 are average scores calculated for each subscale using the factor loadings reported by Vanstone et al. (2015). We divided the weighted sum of items for each scale by the sum of weights to obtain a number ranging between 1 and 5.

Table 1

| Subscale | Mean | Standard Deviation |
| --- | --- | --- |
| Music in Daily Life | 4.09 | 0.71 |
| Emotional Listening Experience | 4.31 | 0.58 |
| Musical Performativity | 3.33 | 1.01 |
| Musical Consumer Behaviour | 3.57 | 0.81 |
| Responsive Music Listening | 3.76 | 0.80 |
| Musical Preference | 4.23 | 0.64 |

Rentfrow and Gosling (2003) propose 4 music preference dimensions. On their Short Test of Music Preferences (Rentfrow & Gosling, 2003) participants are asked to rate on a scale from 1 to 7 how much they like each of 14 music genres, with 1 corresponding to “Not at all”, and 7 corresponding to “A great deal”. The 4 music preference dimensions are as following: reflexive and complex, intense and rebellious, upbeat and conventional, energetic and rhythmic. The values in Table 2 are average scores calculated for each dimensions using the factor loadings reported by Rentfrow and Gosling (2003).We divided the weighted sum of items for each scale by the sum of weights to obtain a number ranging between 1 and 7.

Table 2

| Preference Dimension | Mean | Standard Deviation |
| --- | --- | --- |
| Reflexive and Complex | 4.62 | 0.87 |
| Intense and Rebellious | 4.37 | 1.46 |
| Upbeat and Conventional | 4.60 | 1.01 |
| Energetic and Rhythmic | 4.93 | 0.96 |

The Goldsmiths Music Sophistication Index (Müllensiefen et al., 2014) assesses participants’ self-reported musical sophistication. Participants are asked to indicate how much they agree with each of 31 statements on a scale from 1 (“Completely disagree”) to 7 (“Completely agree”). The values in Table 3 are scores calculated for each of six indices (five subscales and a general sophistication index). The five subscales are as following: active engagement, perceptual abilities, musical training, singing abilities, emotions. We report the sums of all items for each index to allow for easier comparison with the data reported by Müllensiefen et al. (2014).

Table 3

| Subscale | Mean | Standard Deviation |
| --- | --- | --- |
| Active Engagement | 40.05 | 9.56 |
| Perceptual Abilities | 48.74 | 6.29 |
| Musical Training | 32.36 | 8.09 |
| Singing Abilities | 31.49 | 7.14 |
| Emotions | 29.95 | 3.80 |
| General Sophistication | 82.89 | 17.23 |
